# RBP2 Induces Epithelial-Mesenchymal Transition in Non-Small Cell Lung Cancer

**DOI:** 10.1371/journal.pone.0084735

**Published:** 2013-12-20

**Authors:** Shikun Wang, Yang Wang, Haijian Wu, Likuan Hu

**Affiliations:** Department of Radiation Oncology, Qilu Hospital of Shandong University, Jinan, Shandong Province, China; Cincinnati Children's Hospital Medical Center, United States of America

## Abstract

RBP2 has been found to actively participate in cancer progression. It inhibits the senescence of cancer cells, mediates cancer cell proliferation and promotes cancer metastasis. It is also essential to drug tolerance. However, the effects of RBP2 on epithelial-mesenchymal transition are still unknown. In this study, we analyzed the effects of RBP2 on epithelial-mesenchymal transition in non-small cell lung cancer. The results showed that RBP2 down-regulated the expression of E-cadherin by inhibiting the promoter activity of E-cadherin and up-regulated the expression of N-cadherin and snail via the activation of Akt signaling, and the overexpression of RBP2 induced epithelial-mesenchymal transition in non-small cell lung cancer cells. Our study further indicated thatRBP2 may be a potential target for anti-lung cancer therapy.

## Introduction

Lung cancer is the most common cause of cancer mortality, and its morbidity is increasing worldwide [1]. Non-small cell lung cancer (NSCLC) accounts for 85% of all lung cancer. Unfortunately, many NSCLC patients develop distant metastasis during the early stage of the disease. Moreover, mortality among NSCLC patients is more often caused by metastasis rather than their primary tumors. Therefore, the early detection and prevention of metastasis is a key step in stopping the progression of NSCLC [2]. 

Retinoblastoma binding protein-2 (RBP2) was originally identified as a critical retinoblastoma protein (pRB)-binding protein [3]. In 2007, RBP2 was first found to be a histone demethylase for tri- and dimethylated lysine 4 on histone H3 (H3-K4me2 and H3K4me3) [4,5]. It is widely accepted that histone methylation is very important for the expression of various genes and plays important roles in cancer progression [6-8]. Aberrant methylation contributes to the excessive proliferation of cells and tumorigenesis, and the H3K4me0 state is highly correlated with poor prognosis in breast cancer patients [9]. As a histone demethylase, RBP2 actively takes part in cancer progression. However, unlike other histone-modifying enzymes, RBP2 can directly bind target DNA. It has an AT-rich interaction domain (ARID) that specifically recognizes the DNA sequence CCGCCC [10]. This special DNA sequence is enriched in the promoter regions of the RBP2 target genes. In gastric cancer, RBP2 binds to the promoter regions of the p16^ink4a^, p21^CIP1^ and p27^kip1^ genes to inhibit their expressions and diminish the senescence of cancer cells [11]. In lung cancer, RBP2 binds to the promoter region of p27, cyclin D1 and integrin β1 to mediate cancer cell proliferation and metastasis [12]. In this study, we analyzed the effects of RBP2 on epithelial-mesenchymal transition (EMT) in NSCLC.

## Materials and Methods

### Ethics Statement

Patient information and samples were obtained with written informed consent. Each patient in this study gave written informed consent to publish these case details. The research was approved by the ethics committee of Qilu Hospital. 

### Patients

The lung cancer specimens (n=61) and distant normal lung tissues (n=47, 5 cm from the margin of the lung cancer) were collected from patients with NSCLC in Qilu Hospital from 2007 to 2008. The tissues were stored at -80°C until use. All samples were from patients who had not undergone preoperative radiotherapy or chemotherapy. The pathological staging of the 61 patients was performed according to the tumor-node-metastasis (TNM) staging system [13]. 

### Immunohistochemistry

The tissue specimens were embedded in paraffin. The sections were deparaffinized in xylene and rehydrated in an ethanol gradient. After the antigens were retrieved, the sections were treated with 3% H_2_O_2_ for 10min, followed by 5% bovine serum albumin (BSA) for 30 min. Then, the sections were incubated with primary antibodies against RBP2 (1:250 dilution), E-cadherin (1:200 dilution), N-cadherin (1:200 dilution) or snail (1:200 dilution) overnight at 4°C, and secondary antibodies conjugated to HRP (Santa Cruz Biotechnologies, Santa Cruz, CA, USA) were added for 1 h at 37°C. Visualization of antibody binding was performed using DAB staining. The nuclei were stained with hematoxylin. The immunostaining results were independently assessed by two pathologists. The percentage of the positive cancer cells was estimated according to the following criteria: 0 = no positive cancer cells, 1 = < 10% positive cancer cells, 2 = 10%-35% positive cancer cells, 3 = 35%-75% positive cancer cells and 4= > 75% positive cancer cells. The staining intensity was estimated according to the following criteria: 1 = no staining, 2 = light yellow staining (weak staining), 3 = yellow staining (intermediate staining) and 4 = brown staining (strong staining) [14]. The final score was equal to the area score and the intensity score [15]. A final staining score ≥ 4 was defined as overexpression, and a final staining score < 4 was defined as nonoverexpression [15].

### Cell Culture, RNA Interference and Gene Overexpression

The human lung cancer cell lines A549 and SK-MES-1 and the human bronchial epithelial cell line Beas2B were obtained from the American Type Culture Collection (Manassas, VA, USA). Beas2B and A549 cells were grown at 37°C with 5% CO_2_ in RPMI-1640 (Sigma, Santa Clara, CA, USA) media supplemented with 10% fetal bovine serum (FBS) (Gibico, Carlsbad, CA, USA). The SK-MES-1 cells were grown in MEM (Gibico, Carlsbad, CA, USA) supplemented with 10% FBS. When we analyzed the effects of Akt signaling on the expression of N-cadherin and snail, the A549 cells were treated with 24 μM PI3K inhibitor (LY294002) (Cell Signaling Technology, Danvers, MA, USA) for 24 h. For RNA interference and gene overexpression, the cells were cultured in 6-well plates (1.0 × 10^5^ cells/well) overnight, followed by transfection with 5 μl of RBP2 siRNA (Invitrogen, Carlsbad, CA, USA) or 2 μg of the pcDNA3-HA-RBP2 plasmid (a generous gift from W.G Kaelin) using 5 μl of Lipofectamine 2000 (Invitrogen, Carlsbad, CA, USA); the cells were then cultured for 48 h. The following siRNA sequences were used in this study: RBP2 siRNA1 5’-AAUAUCCAGGGCCUUCAUGUAGCCC-3’; RBP2 siRNA2 5’-UUGUGUACUCGUCAAACUCUACUCC-3’; RBP2 siRNA3 5’-UUAACAUGCCGGUUAUCCAGGCUCU-3’; control siRNA 5’-UUCUCCGAAGGUGUCACGUTT -3’. The RBP2 siRNA that could most effectively deplete RBP2 was used in the following experiments.

### RNA Extraction and Quantitative Real-Time PCR

Total RNA from the tissue specimens and cell lines was isolated using the Trizol reagent (Invitrogen, Carlsbad, CA, USA). cDNA was synthesized using the First Strand cDNA Synthesis Kit (Thermo, San Jose, CA, USA). For real-time PCR analysis, the cDNAs were amplified using SYBR Premix Ex Taq (Takara, Otsu, Shiga, Japan) and were detected using an Applied Biosystems 7500 Real-Time PCR System. Relative gene expression was normalized to the expression of β-actin and was calculated using the 2^(-△△CT)^ method [16]. The following primers were used in this experiment: β-actin forward 5’-ATCGTGCGTGACATTAAGGAGAAG-3’ and reverse 5’-AGGAAGGAAGGCTGGAAGAGTG-3’; RBP2 forward 5’-GCTTGGCAATGGG AACAAAA-3’ and reverse 5’-CCGTTGTCTCATTTGCATGTTAA-3’.

### Western Blot

Total cellular proteins were extracted using Radio Immunoprecipition Assay lysis buffer, and 50 μg of total proteins was used for western blot analysis. The polyvinylidene difluoride membranes were probed with antibodies against β-actin (1 : 1000 dilution), RBP2 (1 : 1000 dilution), E-cadherin (1 : 1000 dilution), N-cadherin (1 : 1000 dilution), snail (1 : 1000 dilution), p-Akt (1 : 1000 dilution) or Akt (1 : 1000 dilution) (Cell Signaling Technology, Danvers, MA, USA), followed by incubation with an anti-rabbit horseradish peroxidase (HRP)-conjugated immunoglobulin G (IgG) (1 : 2000 dilution). The blots were subsequently developed using the enhanced chemiluminescence method (Millipore, Billerica, MA, USA). The β-actin signal was used as a control.

### Immunofluorescence

A549 cells were cultured in a 96-well plate and transfected with RBP2 siRNA for 48 h. Then, the cells were fixed with 4% paraformaldehyde, permeabilized with 0.2% Triton X-100, and incubated with primary antibodies against RBP2 (1 : 250 dilution) or E-cadherin (1 : 200 dilution) for 1 h at 37°C. The cells were then incubated with FITC-conjugated secondary antibodies (Santa Cruz Biotechnologies, Santa Cruz, CA, USA) for 30 min at 37°C. The fluorescent images were captured using a confocal laser scanning microscope.

### Transwell Invasion Assays

Transwell membranes (8 μm pore size, 6.5 mm diameter; Corning, Tewksbury, MA, USA) were pre-coated with matrigel (1 μg/ml, diluted with serum-free RPMI-1640 medium). After RNA interference or gene overexpression treatment for 48 h, 1.0 × 10^5^ cells in 200 μl of RPMI-1640 medium without FBS were plated in the upper chambers. Then, 500 μl of RPMI-1640 medium with 10% FBS was added to the lower chamber. After 12 h, the cells on the upper surface of the transwell membranes were removed by a cotton swab. The cells that invaded through the membrane to the lower surface were fixed with methanol and stained with eosin. The cells were counted under a light microscope.

### Wound Healing Assays

Wound healing assays were performed as previously reported [17]. Briefly, the cells were transfected and cultured for 48 h and grown to a confluent cell monolayer. Then, a linear ‘‘wound’’ was drawn in the cell monolayer with a plastic pipette tip. The cells were incubated at 37°C, and the wound was imaged immediately and monitored after 24 h. The distance between the two margins of the “wound” was calculated. 

### Luciferase Assays

A549 cells were transfected with RBP2 siRNA on day 1 and with the E-cadherin promoter reporter plasmid (Invitrogen, Carlsbad, CA, USA) on day 2. To monitor the transfection efficiency, a Renilla luciferase control reporter plasmid controlled by the thymidine kinase promoter was cotransfected into the cells. Beas2B cells were cotransfected with the pcDNA3-HA-RBP2 plasmid and the above two reporter plasmids. After 48 h, a dual luciferase reporter assay system (Promega, Madison, WI, USA) was used to determine the luciferase activity. The E-cadherin reporter plasmid had the same promoter sequence (-799 to -579) that was previously described [18].

### Statistical Analyses

All statistical analyses were performed using the SPSS17.0 software. The χ^2^ test was used to analyze the relationship between the clinicopathological features and RBP2 expression. Bivariate correlations between RBP2, E-cadherin, N-cadherin and snail were also analyzed by the χ^2^ test. Other statistical analyses were performed using a Student t test. The data are shown as the mean ± SD from 3 independent assays. A statistically significant difference was considered when *P* < 0.05.

## Results

### Relationships between RBP2 and the clinicopathological features of NSCLC

**Figure 1:**
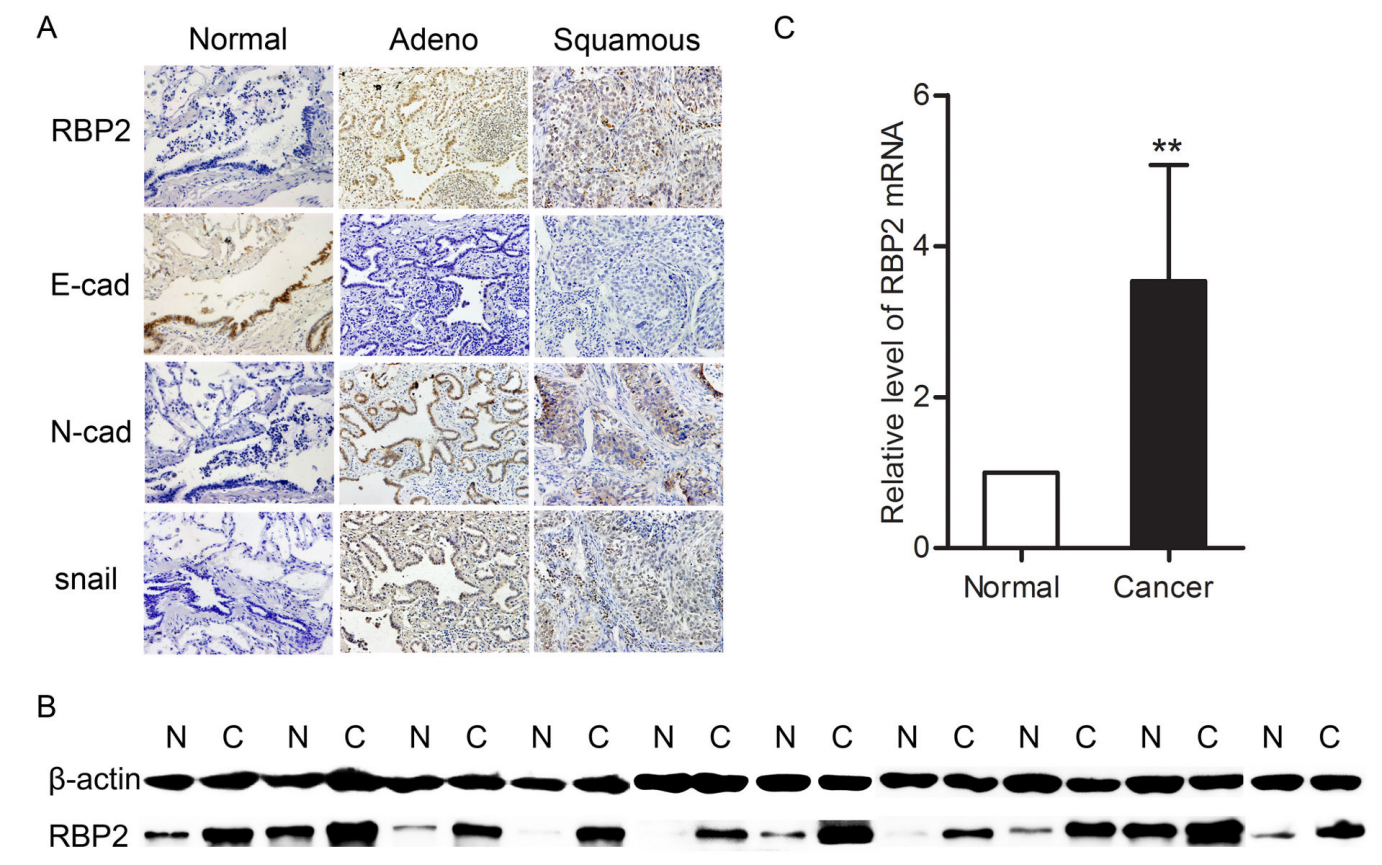
The expression of RBP2, E-cadherin, N-cadherin and snail in normal lung tissues and lung cancer tissues. A. Immunohistochemistrical analysis of RBP2, E-cadherin, N-cadherin and snail (magnification × 200). RBP2, N-cadherin and snail were strongly positive in both adenocarcinoma and squamous carcinoma samples, whereas E-cadherin was weakly stained or unstained. B. Western blot analysis of the RBP2 protein. N = normal lung tissue, C = lung cancer tissue. C. Real-time PCR analysis of RBP2 mRNA. The level of RBP2 mRNA was higher in the lung cancer tissues than in the normal lung tissues (*P* = 0.0011). *P < 0.05 and **P < 0.01.

To study the roles of RBP2 in lung cancer, we first detected the expression of RBP2 in NSCLC tissues and their corresponding normal tissues using immunohistochemistry (Figure 1A). The results were summarized in Table 1. Specimens that overexpressed RBP2 accounted for 52.46% of the cancer samples, and most of these samples exhibited intermediate to strong staining. However, specimens with RBP2 overexpression accounted for only 29.79% of the adjacent normal lung tissues, and most of these samples exhibited weak to intermediate staining. The expression of RBP2 was significantly higher in the NSCLC specimens than that in the normal lung tissues (Table 1). The levels of RBP2 in 10 lung cancer tissues and their corresponding normal lung tissues were further detected using western blot and real-time PCR analyses. The results showed that both RBP2 protein and mRNA were increased in the lung cancer tissues (Figures 1B & 1C).

**Table 1:** Expression of RBP2 in NSCLC tissues and their adjacent normal tissues.

Variable	n	Overexpression* (n)	Overexpression rate (%)	χ^2^	*P*
Cancer tissue	61	32	52.46	5.581	**0.018**
Adjacent tissue	47	14	29.79		

* : A final staining score ≥ 4 was defined as overexpression, and a final staining score < 4 was defined as nonoverexpression.

Then, we analyzed the relationship between RBP2 and the patients’ clinicopathological features, including the patients’ age, gender, pathological type, differentiation and TNM classification. The results showed that there was no clear association between the overexpression of RBP2 and these features (Table 2).

**Table 2:** Correlation of clinicopathological variables with RBP2 protein in NSCLC tissues.

		RBP2 (overexpression*)		
Variable	No. of patients	no	yes	*P*
Age				0.099
≤50 years	32	12	20	
>50 years	29	17	12	
Gender				0.167
Male	33	13	20	
Female	28	16	12	
Pathological type				0.757
Squamous	37	17	20	
Adeno	24	12	12	
Differentiation				0.532
Well	10	6	4	
Moderate	26	13	13	
Poor	25	10	15	
T classification				0.415
T1-2	41	18	23	
T3-4	20	11	9	
N classification				0.533
N0	29	15	14	
N1	32	14	18	

* : A final staining score ≥ 4 was defined as overexpression, and a final staining score < 4 was defined as nonoverexpression.

### Effects of RBP2 on NSCLC cellular migration

**Figure 2:**
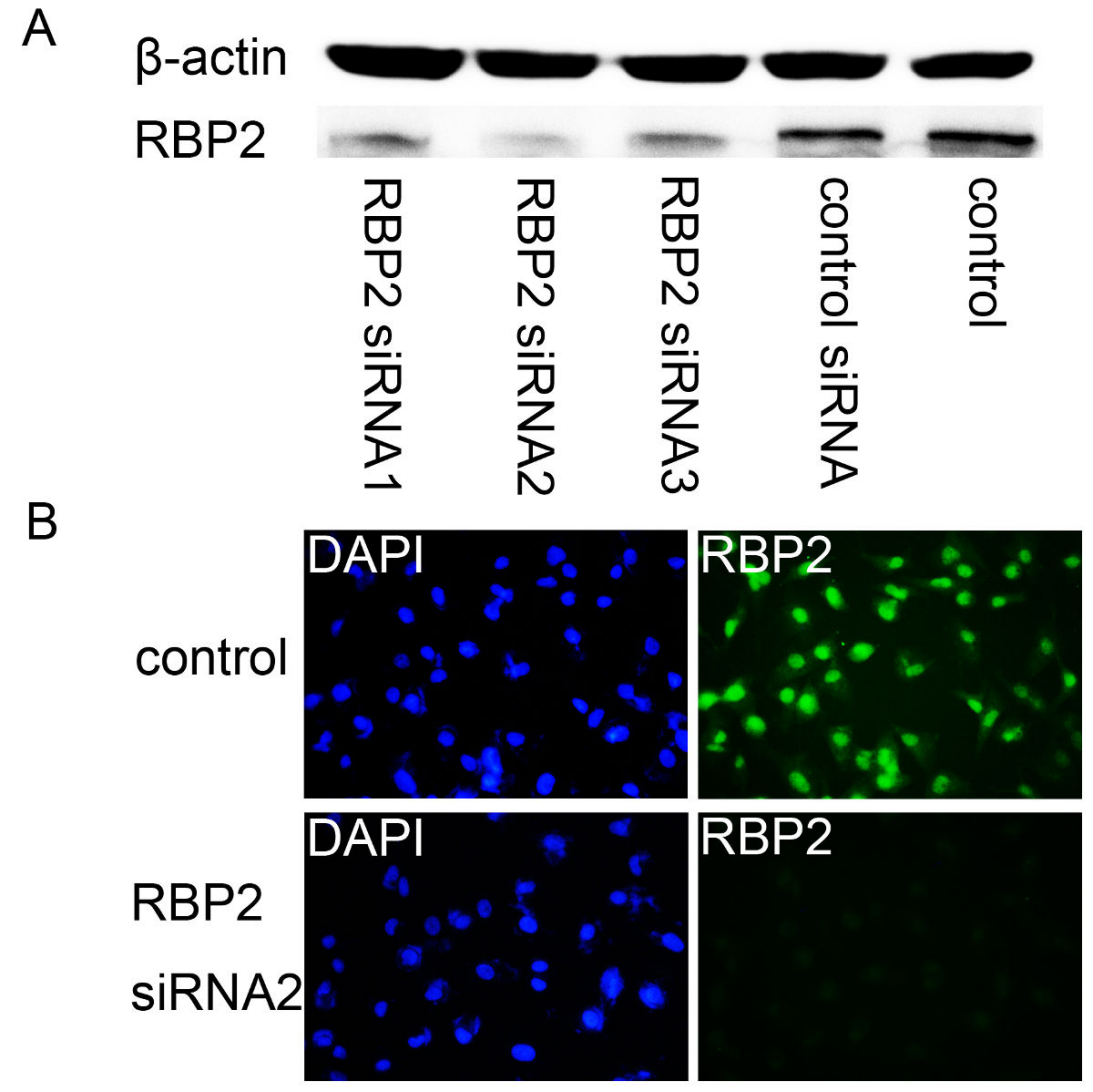
The inhibition of RBP2 by different siRNAs in A549 cells. A. The levels of RBP2 protein in the A549 cells separately treated with different siRNAs. B. Immunofluorescence analysis of RBP2 in untreated A549 cells and cells treated with RBP2 siRNA2 (magnification × 200).

**Figure 3:**
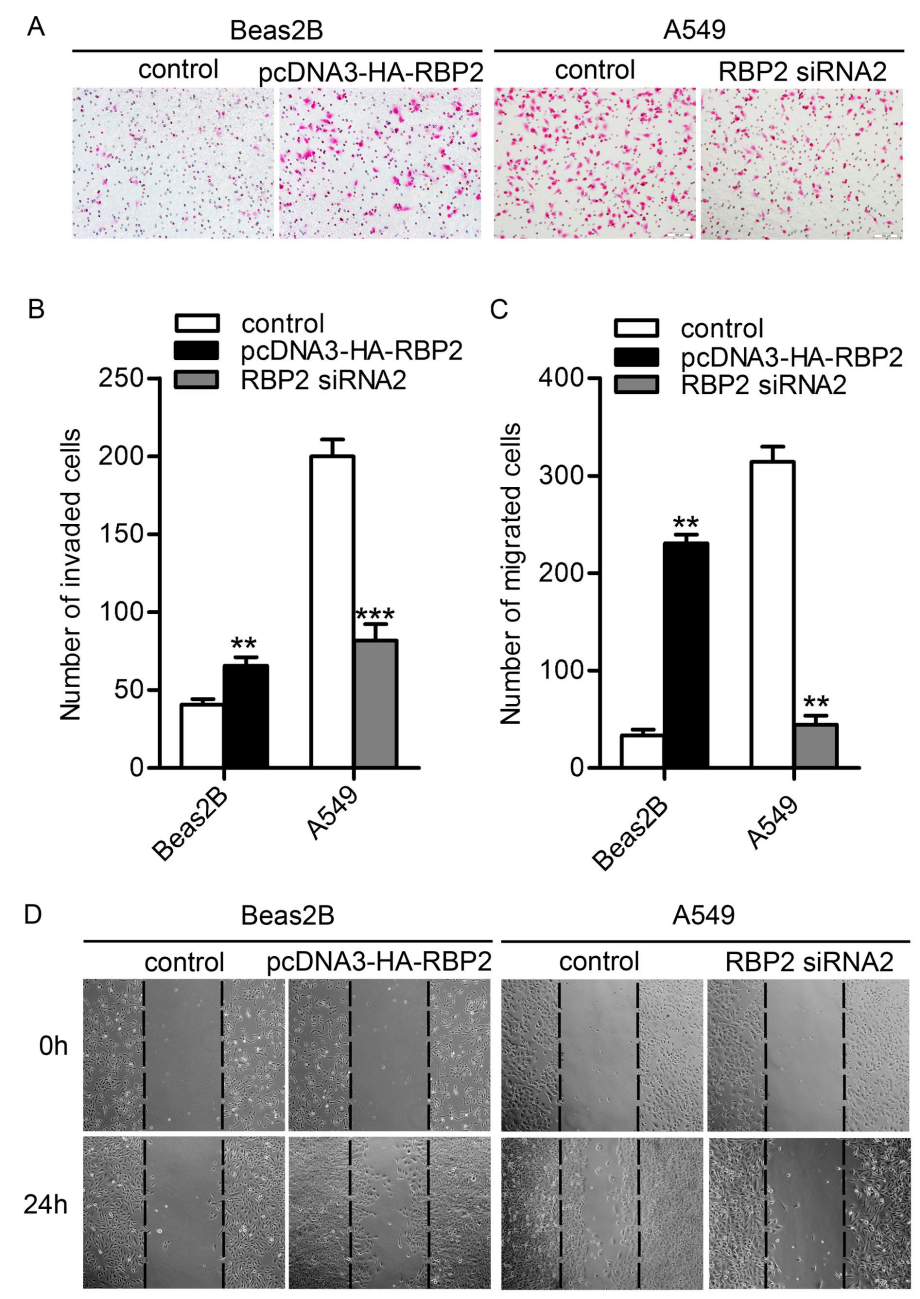
The migratory behaviors of both Beas2B and A549 cells. A. Transwell invasion analysis (magnification × 200). B. The number of invaded cells. More Beas2B cells invaded after transfection with the pcDNA3-HA-RBP2 plasmid (*P* = 0.0011), whereas the number of invaded A549 cells significantly decreased when treated with RBP2 siRNA2 (*P* = 0.0005). *P < 0.05, **P < 0.01 and ***P < 0.001. C. The number of migrated cells. The number of migrated Beas2B cells significantly increased when transfected with the pcDNA3-HA-RBP2 plasimid (*P* = 0.0010), whereas fewer A549 cells migrated into the center space when transfected with RBP2 siRNA2 (*P* = 0.0014). *P < 0.05, **P < 0.01 and ***P < 0.001. D. Wound healing assays (magnification × 100).

To explore the role of RBP2 in lung cancer metastasis, we investigated whether RBP2 regulates cellular migration. First, we detected the inhibition of RBP2 by the three RBP2 siRNAs in A549 cells to choose the most effective siRNA. Western blot analysis showed that the level of RBP2 protein was high in the control A549 cells and decreased after RNA interference of RBP2. Importantly, the RBP2 siRNA2 group exhibited the lowest expression of RBP2 protein (Figure 2A). Furthermore, the expression of RBP2 in the RBP2 siRNA2 group was detected by immunofluorescence analysis. The results showed that RBP2 was negative in the RBP2 siRNA2 group but positive in the control group (Figure 2B). Thus, we chose RBP2 siRNA2 for the depletion of RBP2 in the following experiments. Then, the cellular migratory behaviors of the A549 cells and Beas2B cells were studied using transwell invasion assay and wound healing assay. Compared to the control group, fewer A549 cells passed through the matrigel after deleption of RBP2 while more Beas2b cells passed when RBP2 was up-regulated (Figures 3A & 3B). Consistently, there were fewer A549 cells that migrated from the “wound” edge to the center space when RBP2 was down-regulated, but the number of migrated Beas2b cells was significantly increased after the overexpression of RBP2 (Figures 3C & 3D). These data indicated that RBP2 likely plays a role in cellular migration.

### Bivariate correlations of RBP2 with E-cadherin, N*-*cadherin or snail in the NSCLC tissues

To investigate the roles of RBP2 in cancer metastasis, we studied the effect of RBP2 on EMT, as EMT is closely correlated with cancer metastasis [19]. First, E-cadherin, N-cadherin and snail were detected in the lung cancer tissues and their corresponding normal lung tissues using immunohistochemistry (Figure 1A). The rate of the samples that overexpressed E-cadherin decreased in the cancer tissues compared to their adjacent normal tissues (normal vs cancer, 80.85% vs 40.98%, *P* < 0.01), but the rate of the samples that overexpressed N-cadherin or snail increased in the cancer tissues (normal vs cancer, N-cadherin, 12.77% vs 72.13%, *P* < 0.01; normal vs cancer, snail, 17.02% vs 68.85%, *P* < 0.01). Subsequently, the bivariate correlations of RBP2 with each of these three proteins were analyzed by the χ^2^ test. The results showed that the expression of RBP2 was significantly inversely correlated with the expression of E-cadherin (Table 3), but there was no significant association between RBP2 and N-cadherin or snail (Table 3). 

**Table 3:** Correlations between RBP2 and E-cadherin, N-cadherin and snail in NSCLC tissues.

		RBP2 (overexpression*)		
Variable	No. of patients	no	yes	*P*
E-cadherin				**0.032**
overexpression*	25	16	9	
nonoverexpression*	36	13	23	
N-cadherin				0.095
overexpression*	44	18	26	
nonoverexpression*	17	11	6	
Snail				0.276
overexpression*	42	18	24	
nonoverexpression*	19	11	8	

* : A final staining score ≥ 4 was defined as overexpression, and a final staining score < 4 was defined as nonoverexpression.

### Effects of RBP2 on EMT in NSCLC cell lines

**Figure 4:**
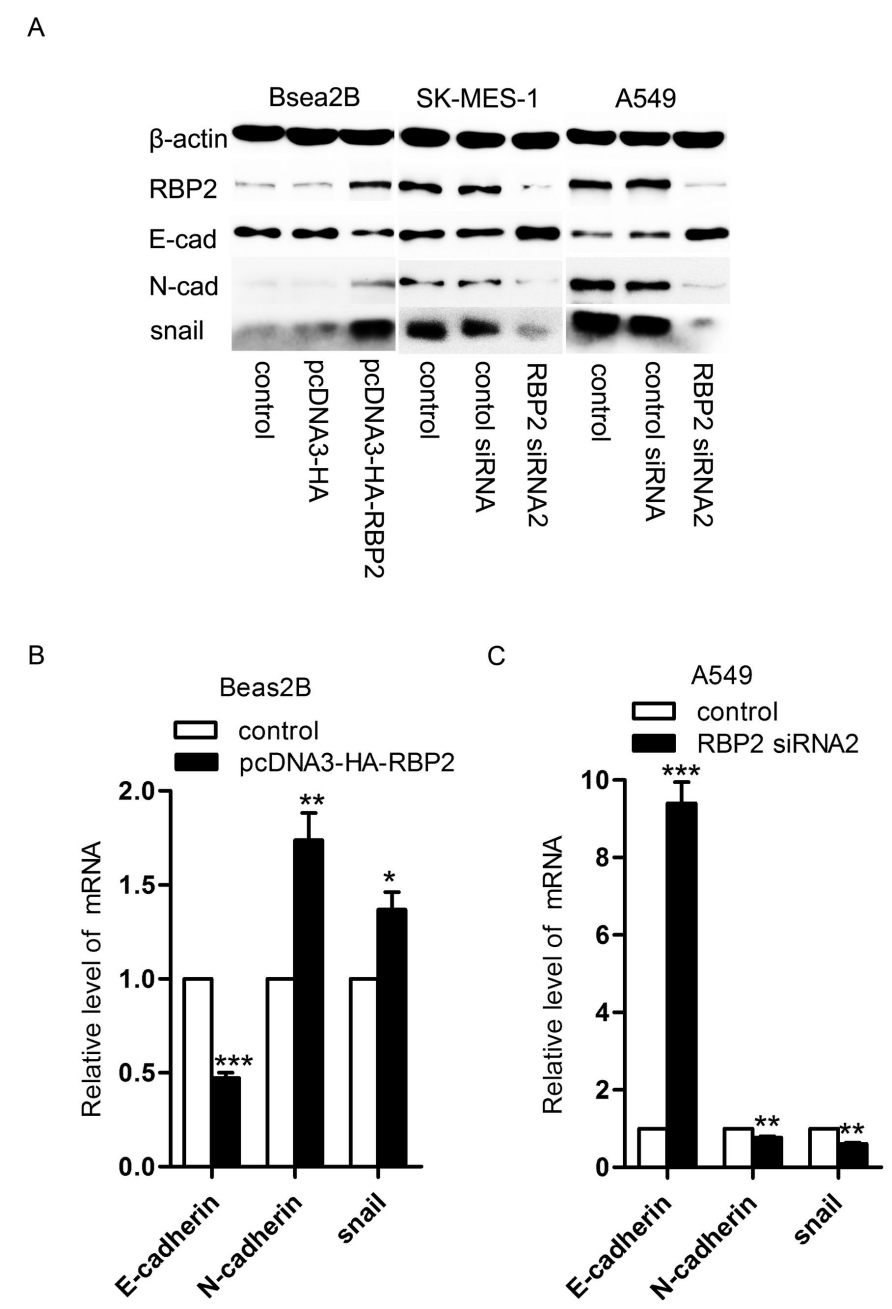
The expression of RBP2, E-cadherin, N-cadherin and snail in both Beas2B and A549 cells. A. Western blot analysis of RBP2, E-cadherin, N-cadherin and snail expression in the Beas2B, SK-MES-1 and A549 cells. When the Beas2B cells were transfected with the pcDNA3-HA-RBP2 plasmid, the levels of RBP2, N-cadherin and snail proteins increased, and the level of E-cadherin protein decreased. When the SK-MES-1 and A549 cells were transfected with RBP2 siRNA2, the levels of RBP2, N-cadherin and snail proteins were increased, and the level of E-cadherin protein decreased. B. Real-time analysis of the levels of E-cadherin, N-cadherin and snail mRNAs in the Beas2B cells. The level of E-cadherin mRNA was higher in the Beas2B cells treated with the pcDNA3-HA-RBP2 plasmid than in the control Beas2B cells (*P* = 0.0005), but the levels of N-cadherin and snail mRNAs were lower (N-cadherin, *P* = 0.0063; snail, *P* = 0.0101). *P < 0.05, **P < 0.01 and ***P < 0.001. C. Real-time analysis of the levels of E-cadherin, N-cadherin and snail mRNAs in the A549 cells. The level of E-cadherin mRNA was lower in the A549 cells treated with RBP2 siRNA2 than in the control A549 cells (*P* = 0.0007), but the levels of N-cadherin and snail mRNAs were higher (N-cadherin, *P* = 0.0037; snail, *P* = 0.0014). *P < 0.05, **P < 0.01 and ***P < 0.001.

To further confirm the effects of RBP2 on EMT, we detected the expression of RBP2, E-cadherin, N-cadherin and snail in the Beas2B cells, A549 cells and SK-MES-1 cells using western blot and real-time PCR analyses. Western blot analysis showed that the expression of RBP2, N-cadherin and snail increased, whereas the expression of E-cadherin decreased in the Beas2B cells transfected with the pcDNA3-HA-RBP2 plasmid (Figure 4A). Meanwhile, both the A549 and SK-MES-1 cells exhibited lower levels of RBP2, N-cadherin and snail proteins and higher level of E-cadherin protein when transfected with RBP2 siRNA2 (Figure 4A). Similarly, real-time PCR analysis further showed that the RBP2, N-cadherin and snail mRNAs increased and the level of E-cadherin mRNA decreased in the Beas2B cells treated with the pcDNA3-HA-RBP2 plasmid (Figure 4B). Additionally, lower levels of RBP2, N-cadherin and snail mRNAs and a higher level of E-cadherin mRNA were observed in both the A549 and SK-MES-1 cells that were treated with RBP2 siRNA2 (Figure 4C).

### Effects of RBP2 on the E-cadherin promoter

**Figure 5:**
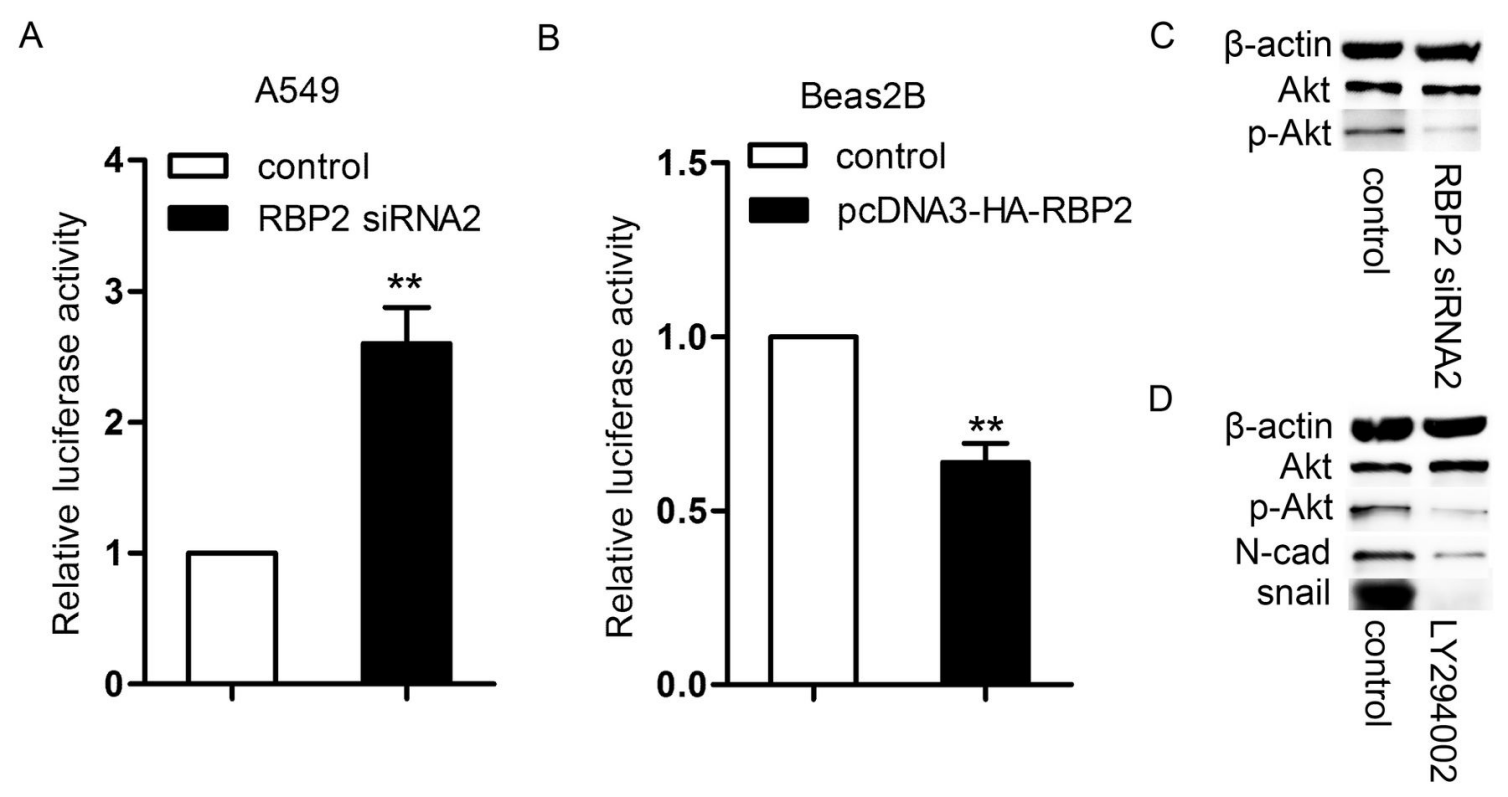
The regulation of E-cadherin promoter activity. A. Eelevated E-cadherin promoter activity in the A549 cells transfected with RBP2 siRNA2 (*P* = 0.0098). *P < 0.05, **P < 0.01. B. The inhibition of the promoter activity in the Beas2B cells transfected with the pcDNA3-HA-RBP2 plasmid (*P* = 0.0075). *P < 0.05, **P < 0.01. C. The expression of p-Akt and Akt proteins in the A549 cells treated with RBP2 siRNA2. D. The levels of p-Akt, N-cadherin and snail proteins in the A549 cells. When A549 cells were treated with LY294002, the expression of these three proteins was reduced.

Because Huang et al. confirmed that RBP2 could directly bind to the promoter region (-799 to -579) of E-cadherin [18] using a CHIP assay, we examined the activity of the same E-cadherin promoter region using a luciferase assay in the A549 and Beas2B cells. When the A549 cells were transfected with RBP2 siRNA2, E-cadherin promoter activity significantly increased (Figure 5A), whereas E-cadherin promoter activity dramatically decreased after RBP2 overexpression in Beas2B cells (Figure 5B). 

### Effects of RBP2 on N*-*cadherin and snail

Both N-cadherin and snail have been shown to be the downstream of the PI3K/Akt pathway [20,21]. Therefore, we hypothesized that RBP2 regulated N-cadherin and snail through the activation of Akt signaling, and we examined the levels of p-Akt and Akt in the A549 cells using western blot analysis. The results showed that p-Akt protein was high in the A549 cells and decreased after the depletion of RBP2 (Figure 5C). The expression of p-Akt was positively correlated with the expression of RBP2. Next, to confirm the effects of Akt signaling on the expression of N-cadherin and snail, we treated A549 cells with a PI3K inhibitor (LY294002) for 24 h and examined the levels of the N-cadherin and snail proteins. LY294002 has been shown to block PI3K-dependent Akt phosphorylation and kinase activity. Interestingly, the levels of both N-cadherin and snail declined (Figure 5D). Therefore, the expression of N-cadherin and snail was positively associated with the expression of p-Akt.

## Discussion

RBP2, a newly identified histone demethylase, belongs to the JARID family and possesses an ARID (A/T rich interaction domain). It often occupies and regulates the promoters of multiple genes that contain the H3K4me3 [5,22]. More importantly, RBP2 is believed to participate in many cell biological functions, especially in tumor biology. Our study reveals a novel insight into the pathophysiology of EMT, and we provide evidence that RBP2 induces EMT in NSCLC. 

RBP2 plays an important role in human cancer. For instance, the overexpression of RBP2 inhibits the senescence of gastric cancer cells [11]. The depletion of RBP2 impairs proliferation but promotes senescence and differentiation in mice lacking Men1 and Rb1 [23]. Drug tolerance of lung cancer cells requires RBP2 [24]. Knockdown of RBP2 led to increased levels of H3K4me3 at the promoters of the DAF and HMOX1 genes in the Beas2B cells [25]. RBP2 up-regulates the expression of cyclin D1, cyclin E1 and integrin β1 to enhance cell proliferation, migration and invasion [12]. In this paper, we also detected the expression of RBP2 in NSCLC tissues and analyzed the relationships between RBP2 and each of the clinicopathological features of NSCLC. The results showed that RBP2 was overexpressed in human NSCLC but there was no significant relationship between the overexpression of RBP2 and each clinicopathological feature. Additionally, the effects of RBP2 on the migration of lung cancer cells were studied, and we found that RBP2 could enhance NSCLC cellular migration. All of these data suggest that RBP2 is an oncogene and provide guidance for cancer treatment.

EMT is a pivotal step in cancer metastasis. During this process, cancer cells derived from epithelial cells lose their epithelial characteristics, such as cellular adhesion, but acquire mesenchymal characteristics, such as cell motility, to escape from the primary tissue and invade the surrounding stroma [26-30]. Epithelial cadherin (E-cadherin), a cell adhesion molecule, is a key molecule of EMT. It plays a vital role in maintaining the epithelial phenotype. The absence of E-cadherin leads to a loss of epithelial morphology [31]. Interestingly, the reduced expression of E-cadherin is often accompanied by increased expression of neural cadherin (N-cadherin) [32]. N-cadherin has been shown to weaken cell adhesion and promote breast cancer cell invasiveness [32,33]. Snail is another important molecule of EMT. It has been confirmed that snail overexpression enhances cancer invasion by promoting cell motility [34]. In this experiment, RBP2 was shown to up-regulate the expression of E-cadherin and down-regulate the expression of N-cadherin and snail. These results indicate that RBP2 is able to induce EMT. Recently, Teng et al. confirmed that RBP2 promoted cancer metastasis by activating integrin β1 [12]. Here, we present a new mechanism that RBP2 may play a role in cancer metastasis by inducing EMT.

E-cadherin has been well demonstrated to be a key mediator of EMT, and disruption of E-cadherin is proved to be a cause of EMT. For example, Cheng et al. and Masszi et al. found that TGF-β1 was unable to induce EMT in the presence of cell-cell contact [35,36]. Masszi et al. even concluded that the absence of E-cadherin-mediated cell-cell contact was permissive for the induction of EMT. Consistent with Masszi et al., Zheng et al. reported that TGF-β1 could not induce EMT in confluent tubular epithelial cells, but that the overexpression of E-cadherin in fibroblasts induced mesenchymal-epithelial transition and inhibited EMT in tubular epithelial cells [37]. Several transcription factors have been shown to control EMT by suppressing E-cadherin promoter activity and repressing E-cadherin expression [38,39]. In this paper, we investigated whether RBP2 induced EMT by suppressing E-cadherin promoter activity and inhibiting E-cadherin expression. Because Huang et al. confirmed that RBP2 directly bound to the E-cadherin promoter region (-799 to -579) [18], we examined the activity of the same E-cadherin promoter region and found that RBP2 indeed weakened E-cadherin promoter activity. These results indicate that RBP2 down-regulates the expression of E-cadherin by inhibiting the promoter activity of E-cadherin.

The mechanism by which RBP2 regulates N-cadherin and snail was also studied in this paper. Much evidence points to a critical role of Akt signaling on EMT. For example, the activation of Akt signaling promotes TGFβ- and EGF-dependent EMT [40,41]. Akt can also phosphorylate IKKα to increase snail expression [20]. Biflorin blocked the invasiveness of the cells by down-regulating N-cadherin, most likely via Akt signaling [21]. Here, our observations showed that RBP2 up-regulated the expression of N-cadherin and snail via the activation of Akt signaling, and the Akt inhibitor could block the effects of RBP2 on the expression of N-cadherin and snail.

Recently, Teng et al. did cDNA microarray analysis in NSCLC cells [12]. And they found that 10 genes participating in metastasis were significantly changed after depletion of RBP2, including dihydropyrimidinase-like 3, integrin β1, solute carrier family 7 member 11, solute carrier family 7 member 5, desmoglein 2, sphingosine-1-phosphate lyase 1, collagen type 1 α1, tropomyosin 1α, cell division cycle 42 and protocadherin β10. However, this study did not analyze the effects of RBP2 on E-cadherin, N-cadherin and snail which are important molecules involved in cancer metastasis. Our research enriches the mechanisms of RBP2-mediated cancer metastasis.

Besides, EMT has been shown to result in acquired resistance to gefitinib in NSCLC cells [42]. Meanwhile, RBP2 is essential to gefitinib resistance in NSCLC cells [24]. According to our results, we infer that the RBP2-mediated gefitinib resistance in NSCLC cells may be closely related to the effect of RBP2 on EMT.

Drug therapy is an important treatment for lung cancer. Considering the current poor survival rate, new therapeutic targets are urgently needed. It has been demonstrated that RBP2 is overexpressed in lung cancer, and its expression is highly associated with cancer cell proliferation, invasion, migration and drug tolerance. Our experiments showed that RBP2 could induce EMT in NSCLC. All of these studies indicate that RBP2 is a potential target for anti-lung cancer therapy.
